# Comparison of clinical outcomes between cruciate-retaining and posterior-stabilized total knee arthroplasty in patients with mild to moderate patellofemoral joint osteoarthritis

**DOI:** 10.3389/fsurg.2026.1840494

**Published:** 2026-06-05

**Authors:** Yanfeng Jia, Liqun Xu, Juncai Xu, Shiqi Qin, Boxuan Zhang, Hongwei Bao, Ran Sun

**Affiliations:** 1 Department of Orthopaedic Surgery, Jingjiang People’s Hospital Affiliated to Yangzhou University, Taizhou, Jiangsu, China; 2 Department of Orthopaedic Surgery, Third Hospital of Hebei Medical University, Shijiazhuang, Hebei, China

**Keywords:** anterior knee pain, patellar crepitus, patellofemoral joint osteoarthritis, prosthesis type, total knee arthroplasty

## Abstract

**Background:**

The clinical results of a total knee arthroplasty (TKA) without patellar resurfacing for knee osteoarthritis have been reported; however, not regarding comparison patellofemoral joint (PFJ) clinical outcomes using different prosthesis types. The purpose of this study is to compare clinical and imaging outcomes in patients with mild to moderate PFJ between cruciate-retaining TKA and posterior-stabilized TKA without patellar resurfacing.

**Methods:**

The clinical and imaging outcomes of PFJ were compared between 107 knees (CR group) with CR TKA and 109 knees (PS group) with PS TKA. Radiological evaluations included the hip-knee-ankle angle (HKA), femoral rotation angle (FRA), patellar tilt angle (PTA), Insall–Salvati ratio (ISR), and patellar facet angle. Clinical assessment included the occurrence of anterior knee pain (AKP), patellar crepitus (PC), range of motion (ROM), Western Ontario and McMaster Universities Osteoarthritis Index (WOMAC), and Knee Society Score (KSS).

**Results:**

The incidence of AKP in the PS group was higher compared to the CR group (*p* = 0.028) with no significant differences in terms of the time to the presentation of PC (*P* > 0.05). ROM in PS TKA was better than CR TKA (*p* < 0.05). There was no significant difference in postoperative KSS, WOMAC, HKA, FRA, PTA and ISR (*P* > 0.05) between the two groups.

**Conclusions:**

This study prompt that TKA without patellar resurfacing for the treatment of PFJ osteoarthritis can obtain excellent clinical and radiological early outcomes regardless of the different prosthesis types. CR and PS TKA can achieve similar clinical results. Postoperative AKP may be more common in PS TKA.

**Level of evidence:**

Level III.

## 1 Introduction

Osteoarthritis of the knee includes tibiofemoral and patellofemoral (PFJ) osteoarthritis, with both often coexisting. PFJ osteoarthritis is a type of tricompartmental degenerative joint disease. Injury and degeneration of the cartilage leads to PFJ osteoarthritis. Biomechanical studies showed that PFJ reactive forces can vary from 2.5 to 7.6 times the body weight during the daily activities of a normal knee joint (1). There are various kinds of treatment methods for PFJ osteoarthritis, of which total knee arthroplasty (TKA) has been reported as a relatively successful surgery with few complications during the treatment of older patients (2–4).

The consensus is that total knee arthroplasty (TKA) for end-stage knee osteoarthritis can have good clinical outcomes (3, 4). Patients with knee osteoarthritis often exhibit concurrent PFJ degeneration. TKA is a suitable surgical procedure for patients over 60 years old and for whom other treatments are ineffective. Although there is controversy about whether a patellar replacement should be performed in TKA (5), current studies have shown that good postoperative outcomes can be obtained whether the patellar was resurfaced or not (4, 6). Benjamin M. Zmistowski, P et al. showed that TKA with patellar resurfacing was not beneficial in treating patients with osteoarthritis of the knee without patellofemoral arthritis (7). To our knowledge, the clinical results of TKA without patellar resurfacing for the tibiofemoral joint combined with PFJ osteoarthritis have been reported, but not in terms of comparison to different types of prosthesis (6). Studying the differences in patellofemoral joint outcomes after TKA using different prostheses is vital for how surgeons preoperatively choose the type of prosthesis.

Knee pain, especially anterior knee pain (AKP), is one of the clinical features of PFJ osteoarthritis. Early TKA often combined with a high degree of AKP, is related to the design of the implant as well as the surgical approach. With the advent of newer prostheses and improved surgical approaches, especially the “patella-friendly” femoral prosthesis, the incidence of patellofemoral complications after TKA have significantly decreased. Previous studies have shown that patients with TKA are at risk for AKP with or without patellar resurfacing (8). The purpose of this study was to compare clinical and imaging outcomes in PFJ between cruciate-retaining (CR) TKA and posterior-stabilized (PS) TKA without patellar resurfacing.

We measured via x-ray the patient's preoperative and postoperative ISR, PTA, and HKA and the patient's postoperative FRA using CT. In our study, TKA was performed in all patients with typical PFJ osteoarthritis combined with mild to moderate TFJ osteoarthritis without patellar resurfacing.

We hypothesized that TKA without patellar resurfacing for the treatment of PFJ osteoarthritis could obtain good clinical outcomes and proper PFJ alignment, and maybe there was a little difference in patellar discomfort and radiological evaluations between patients with PS and CR prosthesis.

## 2 Materials and methods

### 2.1 Patients selection

Between January 2020 and May 2022, patients who underwent TKA in our hospital by a single surgeon were recruited and followed up for a minimum of 3 years. The primary inclusion criterion was strict radiographic verification that patients had typical PFJ and tibiofemoral osteoarthritis. Other inclusion criteria included: 1) Primary TKA. 2) age over 50 years at the time of development of painful osteoarthritis; 3) intact knee without evidence of ligamentous instability or injury; 4) knee with varus or valgus less than 15 degrees. The exclusion criteria included: 1) patients with rheumatoid arthritis; 2) patients with other types of inflammatory arthritis; 3) patients who underwent patellar resurfacing; 4) those with pronounced malalignment of the PFJ or severe arthritic changes (Ahlbäck Grade IV); 5) knee revision surgery was performed during the follow-up. The CR and PS prostheses we use have the same patellofemoral trochlear groove design and are patellar compatible. The patients were further divided into the CR group (CR TKA, 107) and the PS group (PS TKA, 109) according to the prosthesis type.

The Ahlbäck radiographic evaluation scale was applied to rate the severity of osteoarthritis, which determined the grade of the severity of osteoarthritis for every single knee compartment, with the range of 0–5 points, according to joint-space narrowing, sclerosis, subluxation, and osteophytes. As the score increases, the osteoarthritis becomes more severe; grade 0 means no arthritis (9). The knees studied had a mean score of 2.04 points for the patellofemoral compartment, 3.91 points for the medial compartment and 2.53 points for the lateral compartment.

Our Institutional Review Board approved this study. All patients were informed of the study protocol in detail. Written and informed consent was acquired from all patients.

### 2.2 Surgical technique

The patients in the CR group TKA were performed using a Mobile-bearing CR total knee system and the patients in the PS group TKA were performed using a Mobile-bearing PS total knee system. The PS group received the PFC Sigma PS prosthesis (DePuy Synthes, Warsaw, IN, USA), and the CR group received the Gemini SL CR prosthesis (Waldemar Link GmbH & Co. KG, Hamburg, Germany). A single experienced surgeon performed all surgeries. The selection of prosthesis type is determined jointly by the surgeon's personal preference and the intraoperative circumstances. All joints were exposed via the medial parapatellar approach, and the measured resection technique was used to cut the bone. As needed, the extension and flexion gap were assessed with standard spacing blocks and balanced with selective soft-tissue releases. The femoral component was positioned with reference to the surgical transepicondylar axis (sTEA) and the tibial component was positioned with reference to the Akagi line. Using the sTEA as an intraoperative rotational reference is more reliable than the PCA in valgus, varus, and neutrally aligned knees (10). The patella was not replaced in all cases of knee arthroplasty, only patellar osteophyte was removed and patellar denervation, and the soft tissue balance was adjusted.

All patients received identical pain control and rehabilitation programs after the operation, among which the multimodal method was designed to avoid injecting anesthetic and advance the patient's postoperative activity time. On the first day after the operation, patients were encouraged to perform isometric quadriceps exercises and straight-leg raises. The patients began to walk with a walker and took active and passive ROM exercises twice a day for 6 weeks. Patients were encouraged to walk without assisted gait 2–3 weeks after the operation.

### 2.3 Clinical outcomes and radiological evaluations

Occurrence of anterior knee pain (AKP), patellar crepitus (PC), range of motion (ROM), Western Ontario and McMaster Universities Osteoarthritis Index (WOMAC) and Knee Society Score (KSS) were evaluated by an independent surgeon who was blinded to the type of prosthesis prior to the operation and at the last follow-up. This study aims to focus on the items related to the evaluation of patellofemoral joint function. Anterior knee pain (AKP) was defined as pain localized to the anterior aspect of the knee reported by the patient during at least one of the following activities: rising from a seated position, climbing stairs, or squatting. The diagnosis was based on patient self-report and clinical chart documentation. The severity of AKP was evaluated using a visual analog scale (VAS) before operation and at the last follow-up. This system graded pain as no pain (grade 0–1), mild (grade 2–3), moderate (grade 4–6), severe (grade 7–8), and worst (grade 9–10) (9). PC was considered to exist when a tactile sense of grinding in the patella region was detected when the knee flexed. Postoperative range of motion (ROM) was assessed as passive flexion using a standard long-arm goniometer by a single assessor. The measurement was performed with the patient in the supine position, and the maximum flexion angle was recorded without active patient effort.

**Figure 1:**
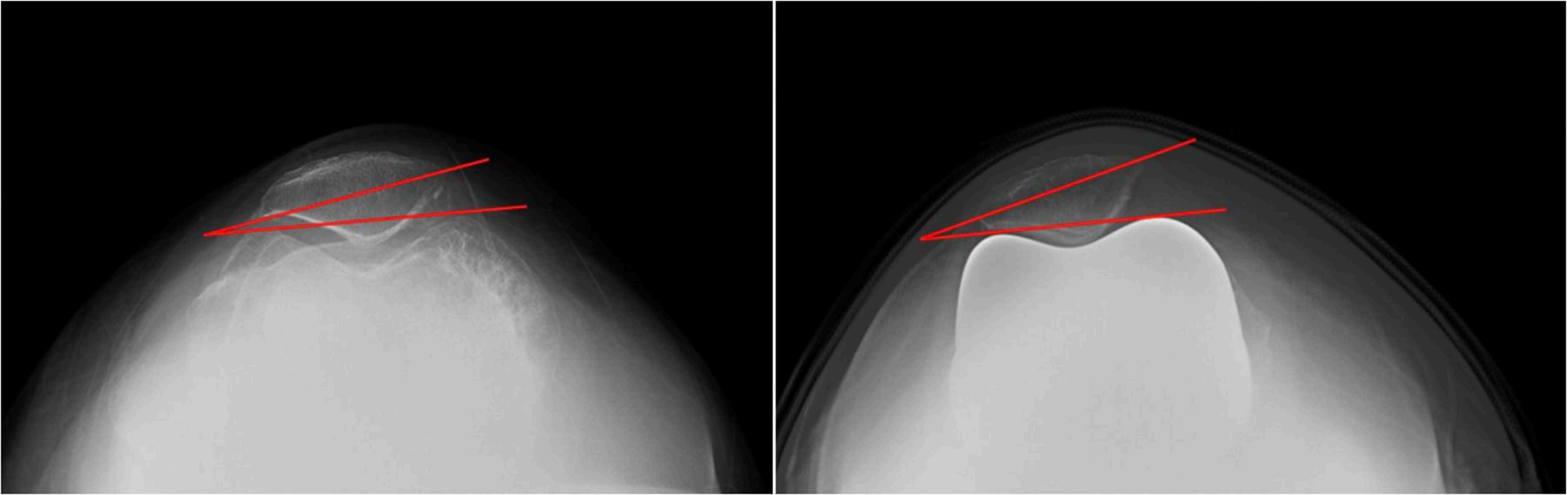
The measurement of patellar tilt angle (PTA). PTA was the angle between the anterior intercondylar line and transverse axis of the patella. The PTA was considered as positive when the transverse axis of the patella tilted laterally from the anterior intercondylar line, and PTA was considered as negative when the transverse axis of the patella tilted medially from the anterior intercondylar line.

**Figure 2:**
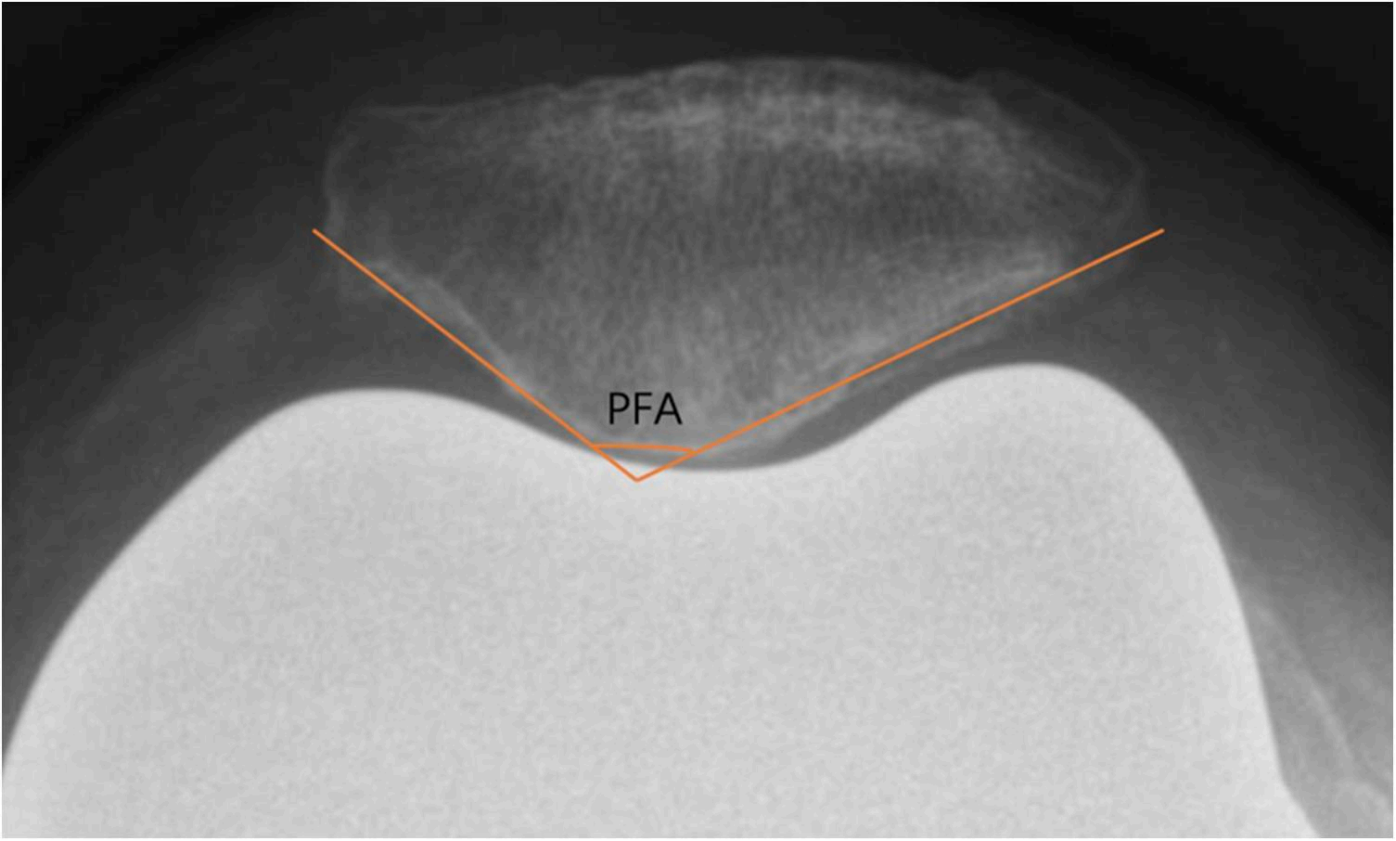
The patellar facet angle (PFA) was indicated by the angle between the medial and lateral facet joint lines.

**Figure 3:**
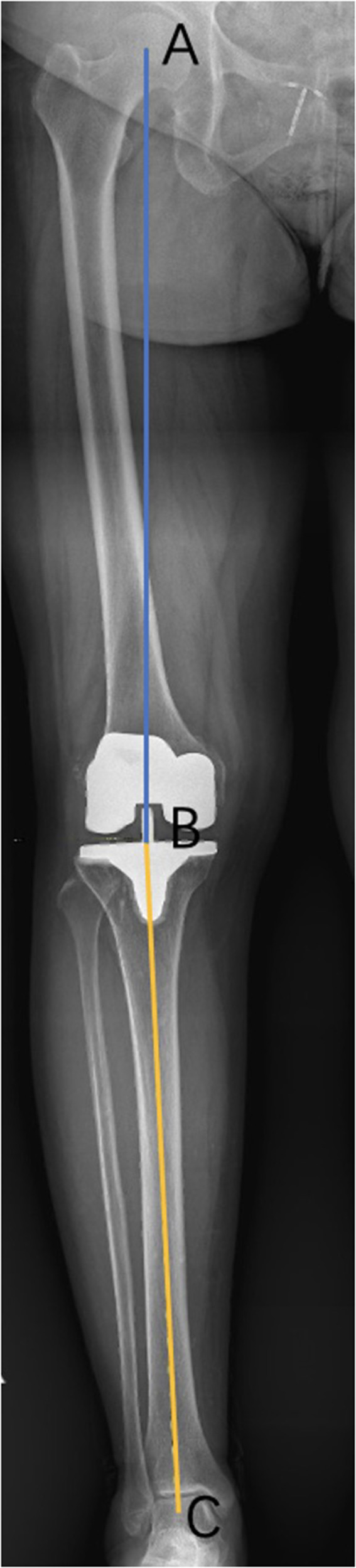
HKA represents the angle formed by the line (AB) from the center of the femoral head to the center of the femoral condyle and the connecting line (BC) from the center of the intercondylar notch to the center of the talus.

**Figure 4:**
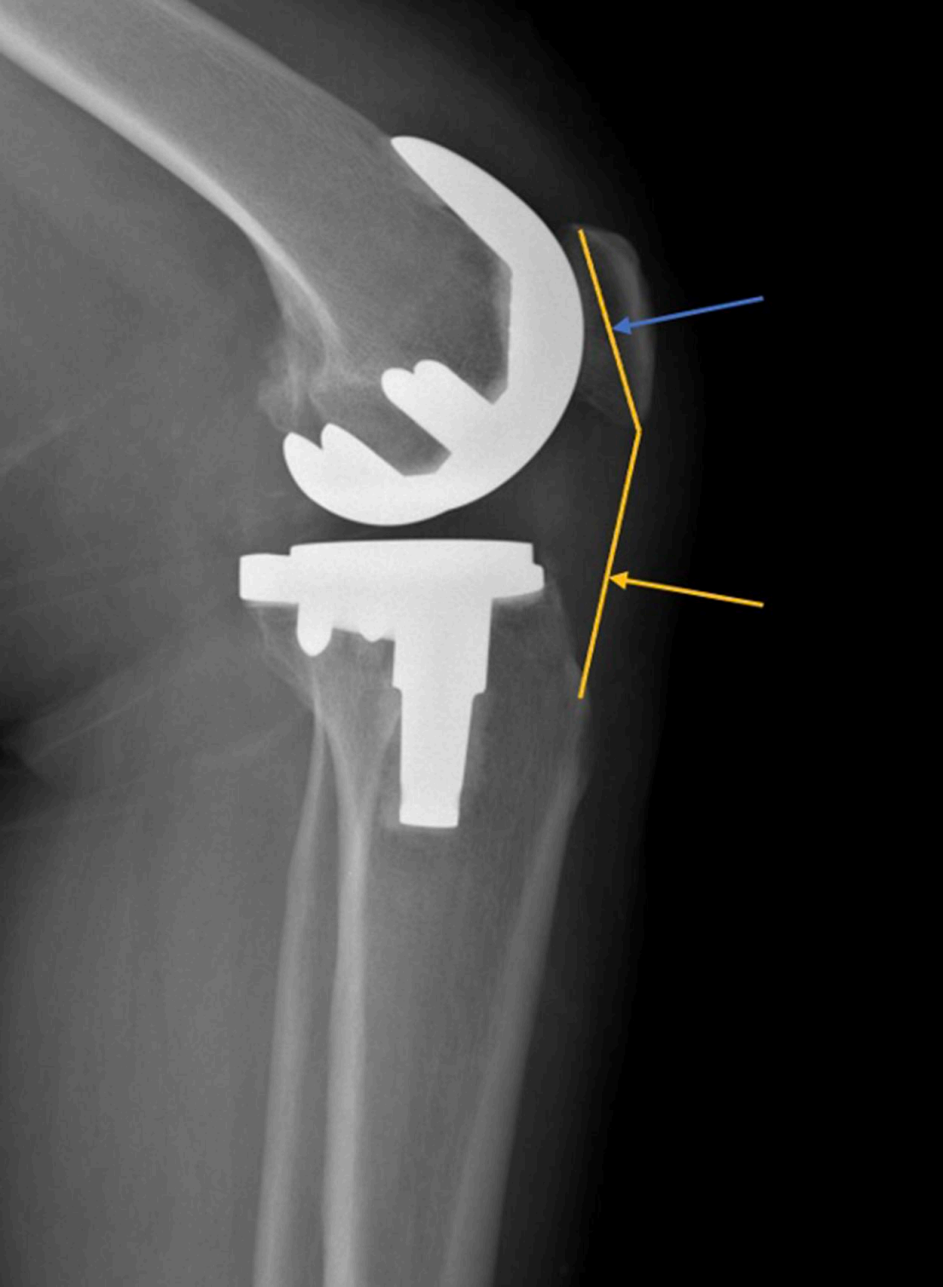
The measurement of insall-salvati ratio (ISR). The ISR was calculated by dividing the patellar tendon length (yellow arrow) by the patella length (blue arrow). Patellar tendon length was the distance between the tibial tuberosity and the inferior pole of the patella. Patella length was the distance between the vertex of the patella and the lowest point of the inner surface of the patella. It was defined as patella alta if ISR > 1.2.

Two independent surgeons blinded to the type of prosthesis evaluated the radiographic parameters. The averaged data from the two surgeons was used in the study. Inter-observer reproducibility was assessed by test–retest analysis of 30 randomly selected radiographs and calculations of an intra-class correlation coefﬁcient (ICC) for all radiographic parameters. For the radiological assessment, the lateral patellar tilt angle (PTA) and patellar facet angle (PFA) were measured using the lateral view radiography with the knee at a 30° flexion, the Insall–Salvati ratio (ISR) was measured using Merchant view, and HKA were measured using a coronal view. The HKA angle was noted as a deviation from 180°. Negative values represent valgus alignment, and positive values represent the varus alignment of the lower limb. A range between +3 and −3 was considered a neutral alignment. PTA was the angle between the anterior intercondylar line and the transverse axis of the patella (Figure 1). We refrained from using the posterior femoral condyle line for PTA measurement due to the reduced data accuracy caused by posterior condyle wear in patients with osteoarthritis. The PTA was considered positive when the patella's transverse axis tilted laterally from the anterior intercondylar line. PTA was considered negative when the transverse axis of the patella tilted medially from the anterior intercondylar line. The PFA was indicated by the angle between the medial and lateral facet joint lines on axial plain x-ray images of the patella (Figure 2). HKA represents the angle formed by the line from the center of the femoral head to the center of the femoral condyle and the connecting line from the center of the intercondylar notch to the center of the talus (Figure 3). HKA is expressed as deviation from 180°. A negative value indicates valgus alignment, and a positive value indicates varus alignment. ISR is the ratio of the length of the posterior surface of the patella tendon to the length of the patella (Figure 4). The ISR was calculated by dividing the patellar tendon length (TL) by the patella length (PL). TL is the distance between the tibial tuberosity and the inferior pole of the patella. PL is the distance between the vertex of the patella and the lowest point of the inner surface of the patella and is defined as patella alta if ISR > 1.2 (Figure 4).

**Figure 5:**
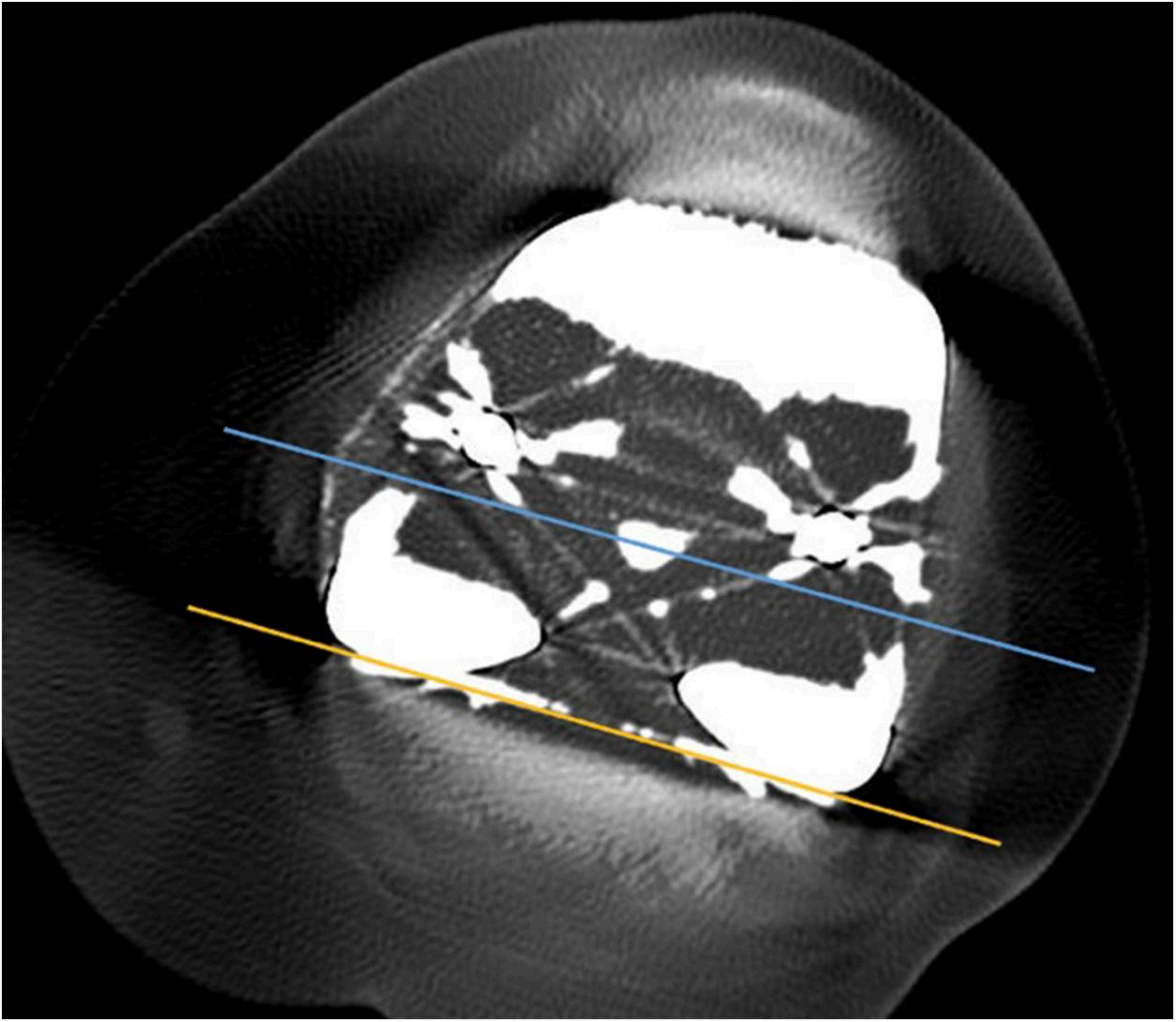
The sTEA (blue line) deﬁned as the line connecting the medial epicondylar sulcus with the most prominent aspect of the lateral epicondyle. Posterior condylar line (yellow line) drawn tangent to the dorsal medial and lateral prosthetic condylar surfaces. The angle between the two lines represents the rotation of the femoral component. The external rotation of the component is positive and the internal rotation is negative.

All patients underwent CT examination postoperative. The CT scans were performed using a Siemens SOMATOM 64-slice CT scanner at our institution. All CT images were reconstructed in the axial, sagittal, and coronal planes. Computer technology was used to reduce the production of metal artifacts. The surgical transepicondylar axis (sTEA) deﬁned as the line connecting the medial epicondylar sulcus with the most prominent aspect of the lateral epicondyle (Figure 5). The angle of femoral component rotation (FRA) was determined by drawing a line tangent to the dorsal medial and lateral prosthetic condylar surfaces and the sTEA. Compared to sTEA, the external rotation of the component is positive, and the internal rotation is negative. FRA can change patellar trajectory and affect patellar symptoms. We measured FRA in all postoperative patients, excluding the effect of femoral component rotation on postoperative results.

### 2.4 Statistical analysis

The data obtained were described as mean ± standard deviation (SD). The Chi-square for independence test was used to compare the differences in AKP and PC. Multivariable logistic regression was not performed due to the limited sample size and low event rate, which would have resulted in model overfitting. The independent-sample *t*-test was used to analyze the differences in clinical and radiological results. To determine the reliability of inter-observer and intra-observer measurements, the intra-class correlation values (ICC) were calculated, and varied from 0 (no agreement at all) to 1 (total agreement). SPSS 25.0 (SPSS, Chicago, IL, USA) was used to analyze data. *P* < 0.05 is defined as statistically significant.

## 3 Results

### 3.1 Demographics

There were 500 TKAs be selected from January 2020 to May 2022, of which 216 TKAs met the inclusion criteria with 107 TKAs undergoing CR TKA and 109 TKAs undergoing PS TKA (Table 1). The CR group had an average age of 68.7 and an average BMI of 25.5. The PS group had an average age of 69.1 and an average BMI of 25.4. The mean follow-up period was 38.0 months for the CR group, and 37.8 months for the PS group. The average Ahlbäck scales in the CR and PS groups were 2.04 and 2.04 for the patellofemoral compartment, 3.91 and 3.90 for the medial compartment and 2.50 and 2.55 for the lateral compartment, respectively. The patients' demographic data were collected with no statistically significant differences between the two groups; the results are shown in Table 1.

**Table 1:** The demographics of the patients.

Characteristics	Group CR	Group PS	*P* value
Knees (n)	107	109	-
Sex (female/male)	83/24	86/23	-
Age	68.7 ± 6.5	69.1 ± 3.0	0.508
Follow-up period (months)	38.0 ± 0.6	37.8 ± 0.7	0.095
Body mass index (kg/m^2^)	25.5 ± 0.9	25.4 ± 0.9	0.734
Ahlbäck scale			
Patellofemoral compartment	2.04 ± 0.36	2.04 ± 0.35	0.972
medial compartment	3.91 ± 0.24	3.90 ± 0.22	0.923
lateral compartment	2.50 ± 0.48	2.55 ± 0.66	0.522

Data are shown as mean ± SD.

### 3.2 Clinical outcomes

**Figure 6:**
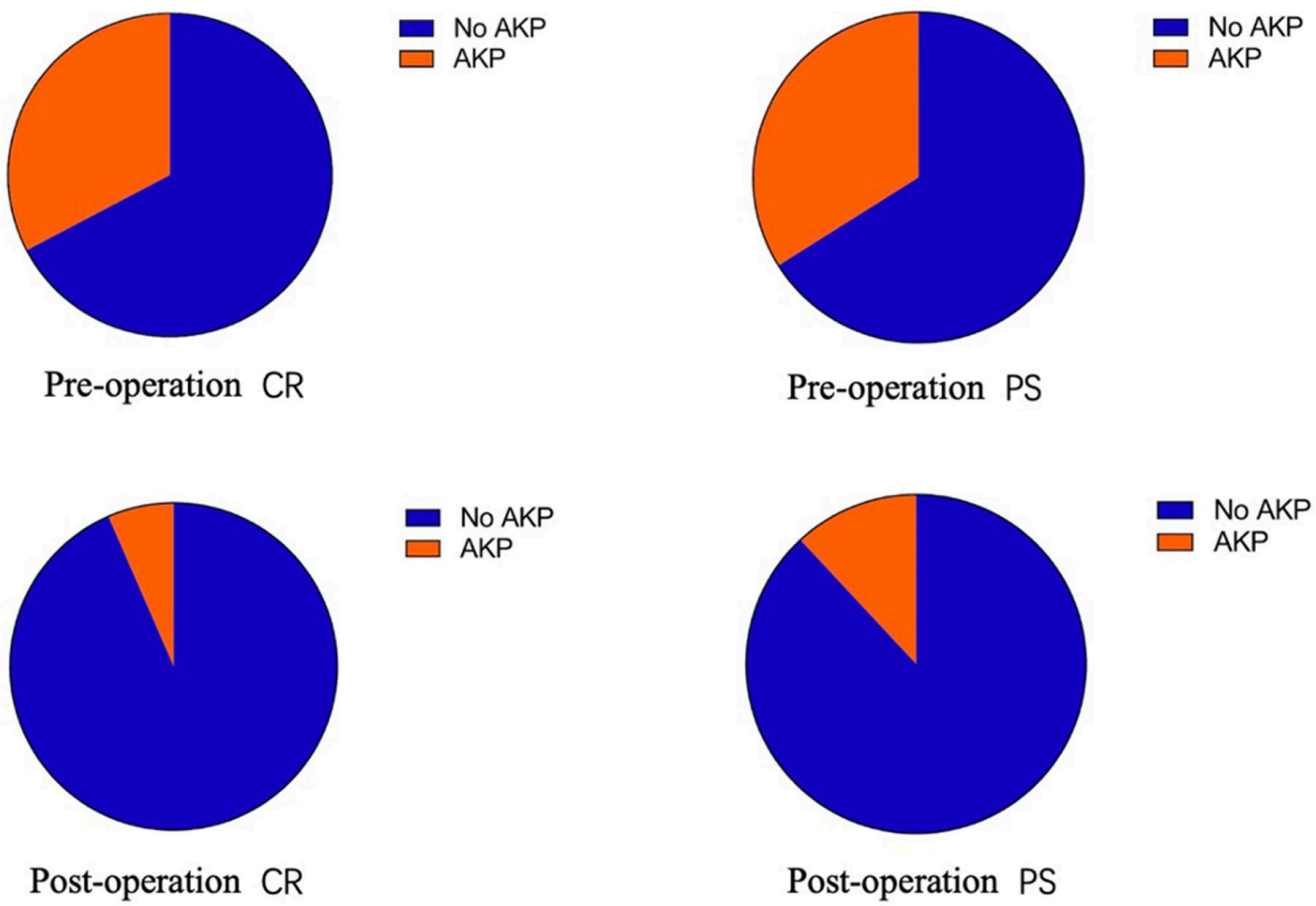
Pie chart showing patients with AKP in the CR and PS groups.

No significant difference was found in preoperative KSS and WOMAC between the CR and PS groups. These parameters were significantly improved in both groups after the operation with no significant difference. The postoperative ROM in the PS group was significantly higher than in the CR group (Table 2). Patients reported AKP in 35 knees in the CR group and 37 knees in the PS group preoperatively (Figure 6). At the final follow up, the AKP was evident in 7 knees in the CR group and 13 knees in the PS group, representing a signiﬁcantly higher rate in the PS group (*p* = 0.028) (Table 3). Among them 7 patients in the CS group were mild, 12 patients in the PS group PS were mild and 1 was moderate. The severity of knee pain was significantly lower in both groups postoperative. The incidence of postoperative AKP in the PS group was higher than in the CR group. After the operation, the average flexion ROM in the PS group was 111.9, and the average flexion ROM in the CR group was 108.4 (*p* < 0.05).

**Table 2:** Pre- and post-operative KSS, WOMAC, ROM and category.

Functional measure	category	Group CR	Group PS	*P* value
Pre-operation				
KSS		46.3 ± 2.5	46.9 ± 2.6	0.051
WOMAC	Total	35.1 ± 1.3	35.5 ± 1.7	0.384
	Pain	34.8 ± 1.1	35.0 ± 1.8	0.409
	Function	31.0 ± 2.6	31.2 ± 2.3	0.771
	Stiffness	38.8 ± 1.6	40.1 ± 2.3	0.089
ROM	flexion (°)	81.8 ± 5.7	81.9 ± 4.1	0.867
	extension (°)	6.0 ± 1.0	6.0 ± 1.2	0.903
Post-operation				
KSS		89.5 ± 5.2^a^	90.0 ± 5.6^a^	0.509
WOMAC	Total	76.3 ± 2.8^a^	76.2 ± 3.5^a^	0.969
	Pain	79.5 ± 3.1^a^	79.7 ± 2.8^a^	0.646
	Function	73.7 ± 2.9^a^	73.6 ± 2.7^a^	0.640
	Stiffness	71.5 ± 3.4^a^	71.7 ± 3.9^a^	0.843
ROM	flexion (°)	108.4 ± 4.7^a^	111.9 ± 4.6^a^	< 0.001
	extension (°)	2.0 ± 1.1^a^	1.9 ± 1.1^a^	0.807

KSS Knee Society Score; WOMAC Western Ontario and McMaster Universities Osteoarthritis Index; ROM range of motion.

a Were significantly improved than that parameter pre-operation.

Data are shown as mean ± SD.

**Table 3:** The AKP and PC.

Parameters	Group CR	Group PS	*P* Value
Pre-operative AKP	35	37	0.925
No pain	72	72	
Mild	11	12	
Moderate	15	18	
Severe	7	6	
Worst	2	1	
Post-operative AKP	7	13	0.028
No pain	100	96	
Mild	7	12	
Moderate	0	1	
Severe	0	0	
Worst	0	0	
PC	9	12	0.052
Average time to presentation of PC (months)	6.1 ± 1.2	6.5 ± 1.1	0.459

AKP, anterior knee pain; PC, patellar crepitus.

Data are shown as mean ± SD.

PC occurred in 9 knees in the CR group and 12 knees in the PS group, indicating that PC occurred more frequently in the PS group PS than in the CR group, but it lacks statistical significance (*p* = 0.052). There was no significant difference in terms of time to the presentation of PC in both groups, with an average of 6.1 months in the CR group and 6.5 months in the PS group (*P* > 0.05) (Table 3). All knees with PC were observed during follow-up and no patella clunk syndrome or other complications were observed.

### 3.3 Radiological evaluations

No significant difference was found in preoperative PTA, HKA, and ISR. There was no significant between-group difference in postoperative PTA, HKA, ISR, PFA, and FRA. There were 17 knees with PFA < 126° in the CR group and 16 knees in the PS group. PFA < 126° in six of the seven knees with AKP in the CR group and ten of the thirteen knees with AKP in the PS group. There were 11 knees with FRA > 5° in the CR group and 13 knees in the PS group. PC was present in 10 of the knees with FRA > 5°, including 4 in the CR group and 6 in the PS group. AKP was present in 11 of the knees with FRA > 5°, including 4 in the CR group and 7 in the PS group. The average postoperative HKA angle in the CR group was 177.6° and 177.9° in the PS group (Table 4).

**Table 4:** The ISR, HKA, FRA, PFA and PTA.

Variables	Group CR	Group PS	*P* value
Pre-operation			
ISR	1.07 ± 0.12	1.04 ± 0.08	0.649
PTA (°)	8.6 ± 2.1	8.5 ± 2.0	0.775
HKA (°)	169.1 ± 5.2	169.0 ± 6.1	0.522
Post-operation			
ISR	1.06 ± 0.10	1.05 ± 0.08	0.885
PTA (°)	9.0 ± 2.0	8.9 ± 2.0	0.660
HKA (°)	177.6 ± 1.9	177.9 ± 1.7	0.504
PFA (°)	128.4 ± 2.8	128.8 ± 2.6	0.347
FRA (°)	3.12 ± 0.35	3.11 ± 0.34	0.883

ISR, Insall–Salvati ratio; PTA, patellar tilt angle; HKA, hip-knee-ankle angle; FRA, femoral rotation angle; PFA, patellar facet angle;.

Data are shown as mean ± SD.

## 4 Discussion

Osteoarthritis refers to the failure to repair of the joint injury due to stress caused by abnormal joint or periarticular tissue (3). In addition, knee cartilage degeneration is more common in the elderly than in young people (11). With the deterioration and deficit of the PFJ surface, pain and functional limitations may gradually appear, impairing the quality of life. Research has shown that approximately 78% of osteoarthritis accelerates the development of chondral and cartilage degeneration. Furthermore, the development of post-traumatic PFJ osteoarthritis could not be prevented by only correcting instability (12). Severe osteoarthritis can lead to disability. Total knee arthroplasty (TKA) is an effective treatment for advanced severe osteoarthritis, which can relieve knee pain and significantly improve the quality of life. More than 50,000 people receive TKA every year in the UK, and 10% of these patients are affected by isolated PFJ osteoarthritis and have the choice of receiving TKA or patellofemoral arthroplasty (13). In this study, patients with typical PFJ osteoarthritis symptoms, consisting of upstairs pain, squat pain, and patellar discomfort, were recruited. Meanwhile, most patients had osteoarthritis complications of other compartments.

The results of the present study demonstrate good clinical and imaging outcomes in knees with patellofemoral osteoarthritis treated with TKA without patellar surface replacement, which is consistent with previous studies by Smith AJ and Beaupre L (14, 15). Joseph A, et al. and Aaron J's, et al. study concluded that patellar surface replacement has a lower rate of secondary patellar surgery than non-patellar surface replacement (6, 45). Therefore, we believe this may be due to the tendency of surgeons to treat patients with patellofemoral-related symptoms when they present after non-patellar surface replacement with a secondary surgical approach. Sophie Putman, et al. considers that when no cause is identiﬁed, non-operative treatment is the best option given the uncertain outcomes of the various available surgical procedures (16). The patellofemoral arthritis does not affect the clinical and imaging results of TKA (2).

In most cases, patients with PFJ osteoarthritis complain that knee pain affects their daily life, with some patients losing their ability to work because of severe pain and dysfunction. Anterior knee pain (AKP) is a common symptom of PFJ osteoarthritis. The etiology of anterior knee pain is multifactorial, involving tendonitis, synovitis, plicae, neuroma, patellar tilt, and overuse of the knee joint. However, patients with PFJ osteoarthritis differ from those with generalized knee osteoarthritis because PFJ osteoarthritis symptoms and dysfunction are not always consistent with the degree of radiographic osteoarthritis. In patients with severely advanced knee osteoarthritis, non-surgical treatment has little effect and can only partially relieve symptoms. The most effective treatment is surgical intervention. A common complication after TKA for osteoarthritis is anterior knee pain. There are many reasons why patients develop AKP: subclinical infection, patella maltracking, midﬂexion instability, and component malrotation. There is a certain incidence of AKP after TKA regardless of patellar replacement. Laskin, et al. reported that the incidence of AKP after TKA was different; however, 6%–25% of patients with patellar retention had experienced AKP after TKA (2). Shervin, et al. reported that AKP appeared in 4%–40% of all patients independent of patellar replacement (17). Barrack RL, et al. demonstrates that postoperative AKP may be related to prosthesis design or surgical technique regardless of whether the patella is replaced (14, 18). In our study, the incidence of postoperative AKP was 9.3% and the incidence rate and severity of AKP were lower than before the operation. The incidence of AKP in patients with the PS prosthesis was higher than in patients with the CR prosthesis. The pain may be caused by the increased activity and consequently greater pressure of PS, rather than being attributed to the pulley factor.

It is required that patient-related factors associated with AKP should receive more attention due to the differences in terms of the anatomy, alignment, and kinematics of PFJ. Whiteside, et al. reported that postoperative PFJ alignment and pressure were related to the occurrence of AKP after TKA (19). The choice of prosthesis type can also have an impact on the AKP. For patients without patellar surface replacement, a patella-friendly femoral component design can reduce postoperative AKP (20). Additionally, the influence on the prosthesis in AKP and other modifiable factors can aggravate AKP, including muscle imbalance, negative emotion, pain management, and valgus gait (21). In contrast, Hidenori Tanikawa, et al. suggests that PS TKA patellar pressure is lower than in CR TKA, which may be due to increased posterior displacement of the PS than the CR femoral prosthesis during flexion (22). AKP can persist for an extended period after TKA. Studies now show that patellar denervation could significantly reduce AKP (23). However, few studies have described the differences between knees affected by AKP and those not affected by AKP.

Patellar crepitus (PC) is another common complication after TKA for PFJ osteoarthritis. Ogawa H, et al. studied the incidence and influencing factors of PC in patients after vanguard PS TKA without patellar resurfacing in a retrospective study. The study revealed that PC occurred in 8.5% of patients and the average time of PC onset was 4 months (24). Outerbridge patellar cartilage grade 4 and joint line elevation were associated with PC (25). Martin, et al. reported that quadriceps tendofemoral contact and pressure in the transitional region between the trochear and intercondylar box could be reduced by reducing the width and thickness of the anterior flange of the femoral component and a lower intercondylar box ratio (<0.7). The probability of tendon irritation and subsequent fibro-synovial proliferation on the posterior surface of the quadriceps tendon should be theoretically reduced by the modified prosthesis design, lowering the incidence of PC (26). Dennis, et al. reported that PC occurred within the first year (a mean of 10.9 months) in most cases and is consistent with previous studies that reported an average onset time of PC was 7.8 months and the incidence of AKP was 16.7% in patients with PC (24, 27). For patients without patellar surface replacement, a patella-friendly femoral component design can reduce postoperative PC (20). In our study, the incidence of PC in patients with the CR prosthesis was lower than in patients with the PS prosthesis; however, there was no statistical difference.

The shape of the patella is one of the factors affecting the alignment of PFJ. Because the shape of the patella varies from person to person, a TKA without patellar resurfacing results in an incongruous PFJ (28). The PFJ shape of the femoral component is the same as a specific prosthesis design, but the shape of the patellar articular surface differs among individuals. Takahashi, et al. studied the impact of patellar shape on postoperative PFJ in patients who received CR TKA without patellar resurfacing and showed that it caused a high internal pressure, especially for the component, if the patellar facet formed a sharper angle (29). Inoue, et al. indicated that the lateral patellar tilt angle was larger in patients with a patellar facet angle less than 126° and patellar osteosclerosis was prone to occur after TKA (30). Senioris, et al. studied the congruence of PFJ after TKA without patellar resurfacing. They reported that improper congruence of PFJ was discovered in TKA with patellar shapes classified as a Wiberg type C (31). In this study, we measured the patient's postoperative PFA as a comparison between the groups; however, we did not examine the effects of patellar shape on patient outcomes and found a high correlation between postoperative AKP and PFA. We paid closer attention to the changes in patella tilt angle defined as the influence of patellofemoral alignment on PFJ.

The results of Verhulst, et al. demonstrated that ISR is the most reliable method of measuring patellar height. This study measured the pre-and post-operative ISR of patients to investigate changes in patellar height (32), and was conducted by measuring PTA with ISR representing patellar alignment.The patellar facet angle, FRA, and intraoperative lateral retinacular release have been reported to affect postoperative patellar force lines and symptoms. Our study observed no statistical difference in patellar facet angle and FRA between the CR and PS patient groups. Lateral retinacular release was performed in patients with patellar extrusion and lateral support band tension (30, 33). There was no significant difference in postoperative ISR; this is in line with previous studies (34).

It was reported that PTA was a preoperative factor that affects postoperative PFJ alignment. The position and rotation of the femoral and tibial components are the intraoperative factors affecting postoperative PFJ alignment. Moreover, some patients need lateral retinacular release and realignment of the proximal extensor mechanism before, during, and after TKA. A soft tissue reconstructive procedure is often required during TKA for patients with preoperative patellar tilt and subluxation (35). Patellofemoral congruity affected postoperative PFJ alignment (lateral patellar tilt) and joint pressure. The short and long-term outcomes of TKA were affected by improper patellofemoral congruity (29). This suggests that proper intraoperative soft tissue balance has an impact on postoperative patellofemoral symptoms. Compared to the TKA knees, where the patella tilts 6 degrees laterally in normal knees, the changes after TKA surgery can be explained by the anatomy of the femur and the design of the femoral component. Femoral glide is the primary determinant of patellar position, and the design of the femoral component plays a crucial role in bending angles over 30°, rather than the type of TKA (22). The design of the femoral prosthesis had the greatest impact. Many femoral components have the trochlear flange which aims to ameliorate patellofemoral tracking, avoid the patellar clunk syndrome, and make it more “patella-friendly”. In our study, there was no significant difference in PTA between the two groups of patients.

Excellent rotation of the femoral component is important for good TKA results. Surgical TEA is considered the most accurate bone marker in measuring the rotation of the femoral components (10, 36). In this study, postoperative FRA was measured in both groups and the differences between the groups were not statistically significant. We found AKP in 11 of the 24 knees (45.8％) with FRA > 5° and PC in 10 knees (41.7％), suggesting that poor rotation of the femoral component has a significant impact on patellofemoral joint outcomes. The rotation angle of the femoral component will change the patellar trajectory and patellofemoral joint pressure, which we believe may be related to AKP and PC. Prior to final implantation, surgeons should verify femoral rotation using gap balancing techniques (equalizing the extension and flexion gaps) in conjunction with measured resection. Navigation systems or patient-specific instrumentation (PSI) may further enhance rotational accuracy, particularly in less experienced hands (37).

The decision to resurface the patella during TKA is controversial. Patellar retention offers the advantages of bone preservation and avoidance of resurfacing-related complications—including maltracking, fracture, polyethylene wear, and extensor mechanism disruption (38)—but is associated with AKP and potentially higher revision rates. Importantly, AKP can occur irrespective of whether the patella is resurfaced.

While patellar resurfacing is associated with fewer early patellofemoral symptoms, this benefit diminishes over time and may be counterbalanced by the need for secondary revision of a failed patellar implant (8, 39). In contrast, the results of Smith AJ and Beaupre's study concluded that neither TKA with transpatellar surface replacement had any benefit compared to TKA without surface replacement (14, 15). Our findings show that non-replacement patellar surface TKA for patients with patellofemoral osteoarthritis improves the patients' patellofemoral symptoms. When considering the future patellar revision rate, significant attention should be directed towards primary patellofemoral arthroplasty. Routine patellar resurfacing may reduce the risk of future revision (40), and omitting it during primary TKA could elevate this risk (18). Nevertheless, a recent 7-year comparative study showed no significant difference in outcomes or revision rates between the two approaches (41). The most important influence on the outcome of TKA is good prosthesis design and precise surgical technique, rather than the choice of the CR or PS prosthesis or whether patellar replacement is chosen.

This study had some limitations. First, the time of follow-up was short. A long-term prospective study is needed to confirm our findings. Second, we did not include patellar replacement patients for comparison because we felt that conventional patellar replacement would not provide better outcomes for patients and would be financially burdensome. Third, we did not include patients with severe patellofemoral osteoarthritis (score of 4 or more) in this study, so this trial lacks an outcome study of patients with severe patellofemoral osteoarthritis. However, given the retrospective nature of this study and potential methodological biases, these results should be interpreted with caution, and further prospective studies are warranted to confirm these observations.

## 5 Conclusion

In conclusion, this study suggests that TKA without patellar resurfacing may yield favorable early clinical outcomes in patients with mild to moderate patellofemoral osteoarthritis and severe tibiofemoral osteoarthritis. The findings prompt that CR and PS types obtained similar excellent clinical results and alignment of the patellofemoral joint. Improper rotation of the femoral component may impact the patellofemoral joint. The postoperative ROM in PS TKA was better than in CR TKA. For patients with mild to moderate patellofemoral arthritis accompanied by AKP, we advise the prudent utilization of PS implants, given that the incidence rate of postoperative AKP may be elevated in comparison to that with CR implants.

## Data Availability

The raw data supporting the conclusions of this article will be made available by the authors, without undue reservation.
